# Empagliflozin in acute myocardial infarction in patients with and without type 2 diabetes: A pre‐specified analysis of the EMPACT‐MI trial

**DOI:** 10.1002/ejhf.3548

**Published:** 2024-12-26

**Authors:** Mark C. Petrie, Jacob A. Udell, Stefan D. Anker, Josephine Harrington, W. Schuyler Jones, Michaela Mattheus, Tomasz Gasior, Peter van der Meer, Offer Amir, M. Cecilia Bahit, Johann Bauersachs, Antoni Bayes‐Genis, Vijay K. Chopra, James L. Januzzi, Renato D. Lopes, Piotr Ponikowski, Xavier Rossello, Morten Schou, Shelley Zieroth, Martina Brueckmann, Mikhail Sumin, Deepak L. Bhatt, Adrian F. Hernandez, Javed Butler

**Affiliations:** ^1^ School of Cardiovascular and Medical Sciences, British Heart Foundation Glasgow Cardiovascular Research Centre University of Glasgow Glasgow UK; ^2^ Women's College Hospital and Peter Munk Cardiac Centre, Toronto General Hospital University of Toronto Toronto ON Canada; ^3^ Department of Cardiology (CVK) of German Heart Center Charité; Berlin Institute of Health Center for Regenerative Therapies (BCRT), German Centre for Cardiovascular Research (DZHK) partner site Berlin Charité Universitätsmedizin Berlin Germany; ^4^ Duke University Department of Medicine, Division of Cardiology and Duke Clinical Research Institute Durham NC USA; ^5^ Boehringer Ingelheim Pharma GmbH & Co KG Ingelheim Germany; ^6^ Boehringer Ingelheim International GmbH Ingelheim Germany; ^7^ Collegium Medicum – Faculty of Medicine WSB University Dabrowa Gornicza Poland; ^8^ Department of Cardiology, University of Groningen University Medical Centre Groningen Groningen The Netherlands; ^9^ Heart Institute Hadassah Medical Center &The Hebrew University Jerusalem Israel; ^10^ INECO Neurociencias Oroño Fundación INECO Rosario Argentina; ^11^ Department of Cardiology and Angiology Hannover Medical School Hannover Germany; ^12^ Heart Institute, Hospital Universitari Germans Trias i Pujol, Barcelona, Spain, and Department of Medicine Universitat Autònomoa de Barcelona Barcelona Spain; ^13^ Max Super Speciality Hospital New Delhi India; ^14^ Massachusetts General Hospital, Harvard Medical School Baim Institute for Clinical Research Boston MA USA; ^15^ Duke Clinical Research Institute Duke University School of Medicine Durham NC USA; ^16^ Institute of Heart Diseases Wroclaw Medical University Wroclaw Poland; ^17^ Hospital Universitari Son Espases, Health Research Institute of the Balearic Islands University of the Balearic Islands Palma de Mallorca Spain; ^18^ Department of Cardiology, Herlev‐Gentofte Hospital University of Copenhagen Herlev Denmark; ^19^ Section of Cardiology, Max Rady College of Medicine University of Manitoba Winnipeg MB Canada; ^20^ First Department of Medicine, Faculty of Medicine Mannheim University of Heidelberg Mannheim Germany; ^21^ Mount Sinai Fuster Heart Hospital Icahn School of Medicine at Mount Sinai New York NY USA; ^22^ Baylor Scott and White Research Institute, Dallas, TX, USA, and Department of Medicine University of Mississippi Jackson MS USA

**Keywords:** Acute myocardial infarction, Empagliflozin, Diabetes, Heart failure

## Abstract

**Aims:**

In the EMPACT‐MI trial, empagliflozin reduced heart failure (HF) hospitalizations but not mortality in acute myocardial infarction (MI). Contemporary reports of clinical event rates with and without type 2 diabetes mellitus (T2DM) in acute MI trials are sparse. The treatment effect of empagliflozin in those with and without T2DM in acute MI is unknown.

**Methods and results:**

A total of 6522 patients with acute MI with newly reduced left ventricular ejection fraction (LVEF) to <45%, congestion, or both, were randomized to empagliflozin 10 mg or placebo. The primary endpoint was time to first HF hospitalization or all‐cause death. Rates of endpoints with and without T2DM and the efficacy and safety of empagliflozin according to T2DM status were assessed. Overall, 32% had T2DM; 14% had pre‐diabetes; 16% were normoglycaemic; 38% had unknown glycaemic status. Patients with T2DM, compared to those without T2DM, were at higher risk of time to first HF hospitalization or all‐cause death (hazard ratio [HR] 1.44; 95% confidence interval [CI] 1.06–1.95) and all‐cause death (HR 1.70; 95% CI 1.13–2.56). T2DM did not confer a higher risk of first HF hospitalization (HR 1.22, 95% CI 0.82–1.83). Empagliflozin reduced first and total HF hospitalizations, but not all‐cause mortality, regardless of presence or absence of T2DM. The safety profile of empagliflozin was the same with and without T2DM.

**Conclusion:**

Patients with acute MI, LVEF <45% and/or congestion who had T2DM were at a higher risk of mortality than those without T2DM. Empagliflozin reduced first and total HF hospitalizations regardless of the presence or absence of T2DM.

## Graphical abstract

Glycaemic status and diabetes in EMPACT‐MI. HF, heart failure; LVEF, left ventricular ejection fraction; MI, myocardial infarction.

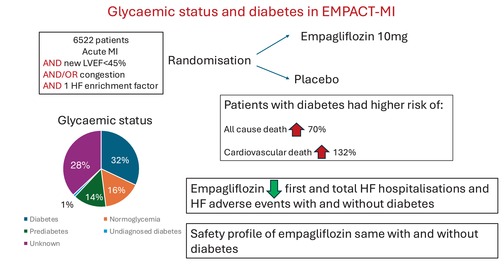

## Introduction

Type 2 diabetes mellitus (T2DM) is increasingly prevalent in patients with acute myocardial infarction (MI). In clinical trials conducted between 10 and 30 years ago, patients with T2DM and acute MI had worse clinical outcomes than those without T2DM.^1^, ^2^ Factors including more advanced epicardial and microvascular coronary disease and impaired healing of the infarct were thought to contribute to higher rates of clinical events among those with T2DM. Contemporary data investigating whether patients with T2DM are still at increased risk in the era of prompt reperfusion and improved medical therapy are sparse.

Sodium–glucose cotransporter 2 (SGLT2) inhibitors reduce the risk of heart failure (HF) hospitalizations in patients with T2DM and chronic stable cardiovascular disease as well as in patients with chronic kidney disease, and patients with HF across the range of ejection fraction, in patients with and without T2DM.^3^ The success of SGLT2 inhibitors in these populations led to interest in their potential efficacy in patients with acute MI. In the SGLT2 inhibitor trials in T2DM and chronic stable coronary disease, patients were excluded if they had suffered an acute MI within 2 months^4^ and few were enrolled within 1 year of an acute MI.^5^ EMPACT‐MI was the first prospective randomized controlled trial to investigate the effect of SGLT2 inhibitors on clinical outcomes after acute MI in a cohort with and without T2DM.^6^ EMPACT‐MI enrolled patients with acute MI who were at a high risk for developing HF. In EMPACT‐MI, empagliflozin did not reduce the primary outcome of first HF hospitalization or all‐cause mortality but reduced first and total HF hospitalizations by 23% and 33%, respectively. DAPA‐MI, which investigated the effect of dapagliflozin on cardiometabolic endpoints in an acute MI population, enrolled only those without T2DM.^7^ Accordingly, a difference between the effect of SGLT2 inhibition in those with or without T2DM and concomitant MI remained unknown. The objective of the current pre‐specified analysis was to compare outcomes of patients with and without T2DM in the EMPACT‐MI trial.

## Methods

### Study design and participants

This was a pre‐specified analysis of the EMPACT‐MI trial. The design, baseline characteristics, and primary results of the double‐blind, randomized, placebo‐controlled, event‐driven EMPACT‐MI trial have been reported previously.^6^, ^8^, ^9^ Briefly, patients were randomized within 14 days of an acute MI who were stable and at high risk for HF based on either newly developed left ventricular systolic dysfunction with documented left ventricular ejection fraction (LVEF) of <45% or signs or symptoms of congestion requiring treatment. Patients were also required to have at least one of the following enrichment factors: age ≥65 years, newly developed LVEF <35%, history of MI, atrial fibrillation, type 2 diabetes, estimated glomerular filtration rate (eGFR) <60 ml/min/1.73 m^2^, elevated natriuretic peptides or uric acid levels, elevated pulmonary artery or right ventricular systolic pressure, three‐vessel coronary artery disease, peripheral artery disease, or no revascularization for the index MI. A total of 6522 participants were randomized in a 1:1 ratio to empagliflozin 10 mg daily or matching placebo on top of standard of care and were followed for a median of 17.9 months. Patients with pre‐existing HF, those who were planned for treatment with an SGLT2 inhibitor and patients with type 1 diabetes were excluded. All participants provided written informed consent and the study protocol was approved by the relevant ethics committee or institutional review board at each participating centre and the coordinating centre.

### Categorization of glycaemic status at baseline

Systematic glycated haemoglobin (HbA1c) measurement during the index acute MI hospitalization was not mandated per protocol. The HbA1c values used in the current analysis were those measured as per clinical practice. For the reporting of baseline glycaemic status, the cohort was grouped into five categories:

1. Normoglycaemia (HbA1c <5.7% [<39 mmol/ml] measured during the index presentation excluding those with baseline diagnosed T2DM).
2. Diagnosed T2DM (all participants with investigator‐reported T2DM regardless of HbA1c measured during the index presentation).
3. Pre‐diabetes (HbA1c ≥5.7% [≥39 mmol/ml] and <6.5% [<48 mmol/mol] measured during the index presentation excluding those with baseline diagnosed T2DM).
4. Undiagnosed diabetes (HbA1c ≥ 6.5% [≥48 mmol/mol] measured during the index presentation excluding those with baseline diagnosed T2DM).
5. Unknown HbA1c (HbA1c not measured during the index presentation excluding those with baseline diagnosed T2DM).

For the purposes of investigation of outcomes and the treatment effect of empagliflozin according to T2DM status, the following categories were used:

1. T2DM: investigator‐reported T2DM plus undiagnosed diabetes (baseline HbA1c ≥6.5%).
2. No T2DM: normoglycaemia plus pre‐diabetes.
3. Unknown HbA1c: HbA1c not measured during the index presentation and no prior diagnosis of T2DM.

### Endpoints

The primary endpoint of EMPACT‐MI was time to first HF hospitalization or all‐cause mortality. The key secondary endpoints were total (first and recurrent) number of HF hospitalizations or all‐cause mortality, total number of non‐elective cardiovascular hospitalizations or all‐cause mortality, total number of non‐elective all‐cause hospitalizations or all‐cause mortality and total hospitalizations for MI and all‐cause mortality. Other outcomes included total number of HF hospitalizations. All events were classified according to pre‐specified definitions by site investigators blinded to study drug assignment and trained on the trial protocol without central adjudication. In supplementary analyses, we also examined site‐reported first and total number of HF adverse events based on the narrow standardized MedDRA query ‘cardiac failure’ alone and as composite with all‐cause mortality. Any adverse events included in a pre‐specified list of cardiac failure events were to be considered always serious and therefore captured. These included both those reported as requiring or prolonging hospitalization (such as prolonged hospitalization due to HF) or reported outpatient HF events as well. The details of this approach in the overall EMPACT‐MI population have been previously published.^10^

Because of the established safety profile of empagliflozin, we used focused safety reporting, in which the investigators reported only serious adverse events, adverse events that led to discontinuation of the trial regimen for at least 7 consecutive days, and adverse events of special interest.

### Statistics

All analyses were performed based on the intention‐to‐treat principle and included all randomized participants. Baseline characteristics were summarized by categories of diabetes at baseline (three group comparison: T2DM, no T2DM, unknown HbA1c) using means (standard deviation) and medians (interquartile range) for continuous variables, and proportions for categorical variables and differences were evaluated using an analysis of variance for continuous variables and the chi‐square test for categorical variables. Among placebo‐assigned patients, we evaluated the risk for the primary endpoints (and its components) and key secondary endpoints for patients with T2DM, and unknown HbA1c versus those with no T2DM. Data for patients who did not have an event were censored on the last day they were known to be free of the outcome. Analyses for time‐to‐first event outcomes were performed based on a multivariable Cox proportional hazards regression model including factors for age, sex, eGFR (assessed categorically using the Chronic Kidney Disease Epidemiology Collaboration formula <45 vs. 45–<60 vs. 60–<90 vs. ≥90 ml/min/1.73 m^2^), geographical region, T2DM, persistent/permanent atrial fibrillation, prior MI, peripheral artery disease, smoking status, and LVEF, and resulting effect estimates were expressed as hazard ratios (HRs) along with their 95% confidence intervals (CIs). Analyses for total (first and recurrent) events were performed based on a negative binomial regression model including the same covariates that were used for time‐to‐first event analyses and including logarithm of observation time as an offset variable. Resulting effect estimates were expressed as rate ratios (RRs) along with their 95% CIs.

Treatment effects of empagliflozin versus placebo were evaluated by T2DM status for the primary outcome and its components, key secondary and other endpoints using Cox proportional hazards regression models for time‐to‐first event endpoints and using negative binomial regression models for total HF hospitalizations. These multivariable models included the same covariates as described above with the addition of a term for treatment and an interaction term between treatment and the subgroup variable to explore potential effect modification of T2DM status. Additionally, treatment effects were evaluated by T2DM status and according to the prescription of metformin or insulin, or according to body mass index (BMI), age or sex at baseline using multivariable models including the same covariates. The effect of empagliflozin versus placebo across the range of baseline HbA1c (where measured) was also evaluated and displayed graphically using a cubic spline model that included a set of cubic polynomials which were constrained to meet at each of a set of equally distanced knots to explore for interaction. Analyses were not adjusted for multiplicity and should be interpreted as exploratory.

Safety outcomes of interest, including hypotension, hypovolaemia, and acute kidney injury were assessed according to T2DM categories. All statistical analyses were performed using SAS software version 9.4 (SAS institute Inc., Cary, NC, USA).

## Results

### Glycaemia status at baseline (over pooled empagliflozin and placebo groups)

**Figure 1:**
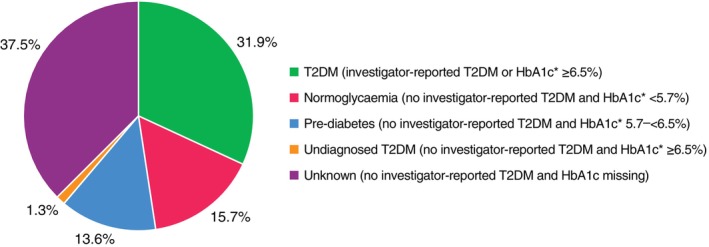
Glycaemic status at baseline. HbA1c, glycated haemoglobin; T2DM, type 2 diabetes mellitus. *During index hospitalization.

Overall, 32% of the participants had T2DM, 14% had pre‐diabetes, 16% were normoglycaemic; 38% had unknown glycaemic status, 1% had undiagnosed T2DM (*Figure* *1*). Patients with T2DM were more likely to be female, to have had a non‐ST‐elevation MI, a history of hypertension and an eGFR <60 ml/min/1.73 m^2^ (*Table* *1*). Patients with T2DM also had a higher BMI and were more likely to be receiving a loop diuretic.

**Table 1:** Baseline characteristics according to type 2 diabetes mellitus status

Characteristic	T2DM (*n* = 2165)	No T2DM (*n* = 1911)	Unknown HbA1c (*n* = 2446)	*p*‐value
Age, years, mean (SD)	64 (10)	63 (11)	64 (11)	0.1141
Sex (%)				
Male	71	78	77	<0.0001
Female	29	22	23	
Index myocardial infarction type, (%)				
STEMI	69	79	76 < 0.0001
NSTEMI	32	21	24	
Race (%)				
Asian	15	15	9	<0.0001
Black or African American	2	1	2	
Other including mixed race	0.2	0.4	0.1	
White	82	79	89	
Medical history (%)				
Hypertension	80	63	65	<0.0001
COPD	6	5	5	0.3373
AF	11	11	11	0.8540
Previous stroke or TIA	6	4	4	0.0140
Previous MI	16	10	13	<0.0001
Previous PCI	15	12	12	0.0005
Previous CABG	3	2	1	0.0004
Smoker (%)				
Never	27	27	26	<0.0001
Current	30	38	35	
Former	44	35	39	
HbA1c^a^, %, mean (SD)	8 (2)	6 (0.4)	NA	<0.0001
NT‐proBNP highest^b^, pg/ml, median (IQR)	1717 (678–3569)	1810 (743–3623)	1944 (867–3710)	0.1689
eGFR, ml/min/1.73 m^2^, mean (SD)	75 (21)	78 (18)	76 (20)	<0.0001
<60 ml/min/1.73 m^2^ (%)	27	18	22	<0.0001
Creatinine, mg/dl, mean (SD)	1 (0.3)	1 (0.3)	1 (0.3)	<0.0001
Haemoglobin, g/dl, mean (SD)	13 (2)	14 (2)	14 (2)	<0.0001
BMI, kg/m^2^, mean (SD)	29 (5)	28 (5)	27 (5)	<0.0001
Systolic BP, mmHg, mean (SD)	123 (15)	118 (15)	121 (15)	<0.0001
Diastolic BP, mmHg, mean (SD)	74 (10)	72 (10)	74 (10)	<0.0001
Medical therapy at baseline (%)				
Beta‐blockers	78	81	74	<0.0001
ACEi or ARB	69	70	66	0.0057
MRA	39	42	37	0.0038
Loop or high‐ceiling diuretics	40	29	33	<0.0001

ACEi, angiotensin‐converting enzyme inhibitor; AF, atrial fibrillation; ARB, angiotensin receptor blocker; BMI, body mass index; BP, blood pressure; CABG, coronary artery bypass graft; COPD, chronic obstructive pulmonary disease; eGFR, estimated glomerular filtration rate; HbA1c, glycated haemoglobin; IQR, interquartile range; MI, myocardial infarction; MRA, mineralocorticoid receptor blocker; NSTEMI, non‐ST‐elevation myocardial infarction; PCI, percutaneous coronary intervention; NT‐proBNP, N‐terminal pro‐B‐type natriuretic peptide; SD, standard deviation; STEMI, ST‐elevation myocardial infarction; T2DM, type 2 diabetes mellitus; TIA, transient ischaemic attack.

^a^ Based on 1201 patients with T2DM who provided data.

^b^ Based on 803 patients with T2DM, 998 patients with no T2DM and 747 patients with unknown HbA1c who provided data.

### Outcomes in the placebo group according to type 2 diabetes status

**Figure 2:**
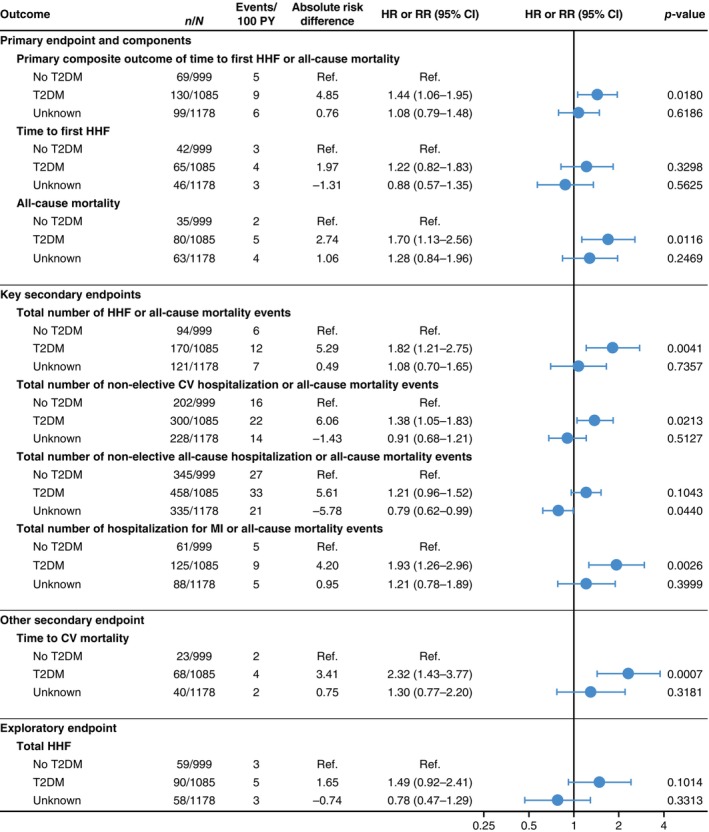
Adjusted event rates in the placebo group according to baseline type 2 diabetes mellitus (T2DM) status. Hazard ratios (HR) and rate ratios (RR) based on Cox regression or negative binomial regression models adjusted for age, sex, estimated glomerular filtration rate (assessed categorically using the Chronic Kidney Disease Epidemiology Collaboration formula <45 vs. 45–<60 vs. 60–<90 vs. ≥90 ml/min/1.73 m^2^), geographical region, T2DM, persistent/permanent atrial fibrillation, prior myocardial infarction (MI), peripheral artery disease, smoking status and left ventricular ejection fraction. T2DM is defined as diagnosed T2DM (investigator‐reported) and undiagnosed T2DM (i.e. baseline glycated haemoglobin ≥6.5%). No T2DM is defined as normoglycaemia or pre‐diabetes. Unknown refers to no T2DM without glycated haemoglobin measured. CI, confidence interval; CV, cardiovascular; HHF, hospitalization for heart failure; PY, patient‐years.

Patients with T2DM, compared to those with no T2DM, were at higher risk of both the primary outcome (HR 1.44, 95% CI 1.06–1.95) and all‐cause mortality (HR 1.70, 95% CI 1.13–2.56) (*Figure* *2* and online supplementary *Figure* *S1*). T2DM did not confer a higher risk of first HF hospitalization (HR 1.22, 95% CI 0.82–1.83).

Type 2 diabetes mellitus conferred a higher risk of three of the four key secondary endpoints: total HF hospitalizations or all‐cause mortality (RR 1.82, 95% CI 1.21–2.75), total non‐elective cardiovascular hospitalizations or all‐cause mortality (RR 1.38, 95% CI 1.05–1.83) and total hospitalizations for MI and all‐cause mortality (RR 1.93, 95% CI 1.26–2.96) (*Figure* *2* and online supplementary *Figure* *S1*). Cardiovascular mortality was higher in those with T2DM (HR 2.32, 95% CI 1.43–3.77), but total HF hospitalizations were not statistically different (RR 1.49, 95% CI 0.92–2.41).

### Effect of empagliflozin according to type 2 diabetes status

**Figure 3:**
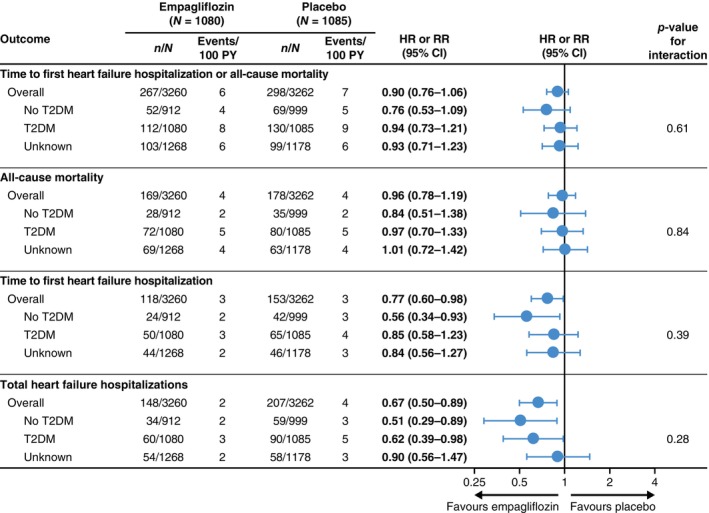
Effect of empagliflozin according to baseline glycaemic status. Hazard ratios (HR) and rate ratios (RR) based on Cox regression or negative binomial regression models adjusted for age, sex, estimated glomerular filtration rate (assessed categorically using the Chronic Kidney Disease Epidemiology Collaboration formula <45 vs. 45–<60 vs. 60–<90 vs. ≥90 ml/min/1.73 m^2^), geographical region, type 2 diabetes mellitus (T2DM), persistent/permanent atrial fibrillation, prior myocardial infarction, peripheral artery disease, smoking status and left ventricular ejection fraction (categorical or continuous). T2DM is defined as diagnosed T2DM (investigator‐reported) and undiagnosed T2DM (i.e. baseline glycated haemoglobin ≥6.5%). *n* number of patients with event (for time‐to‐first event endpoints) or number of events (for total number of events endpoint) based on *N* number of patients at risk. No T2DM is defined as normoglycaemia or pre‐diabetes. Unknown refers to no T2DM without glycated haemoglobin measured. CI, confidence interval; PY, patient‐years.

**Figure 4:**
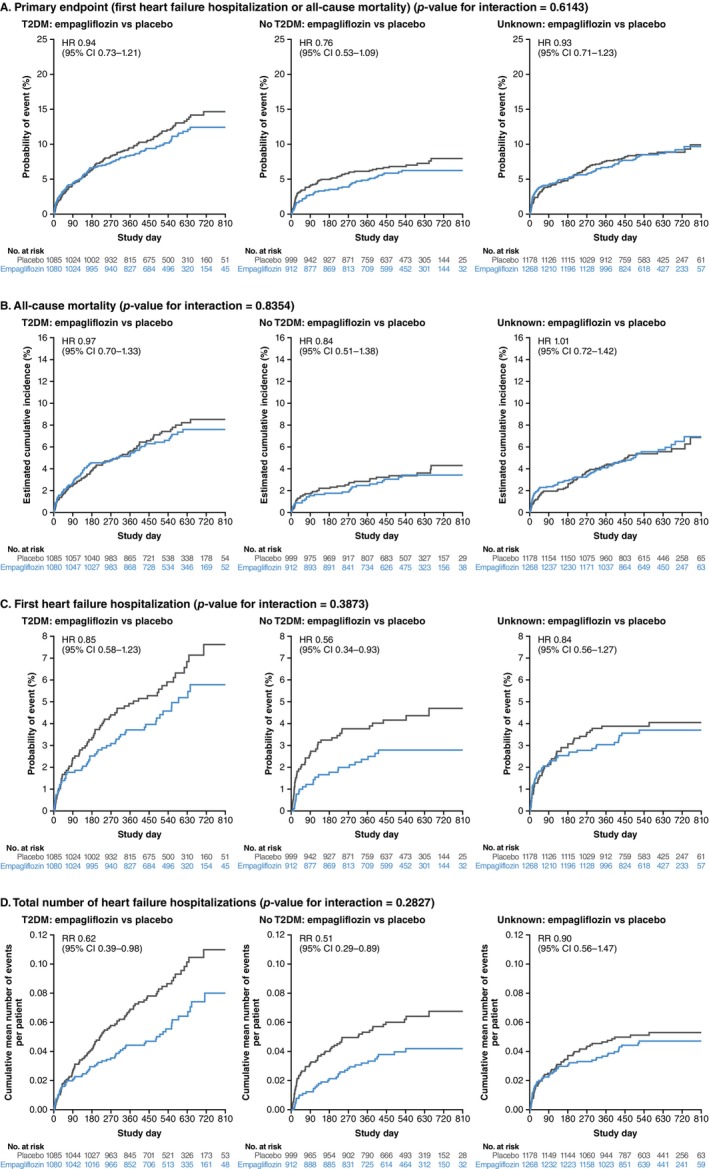
Treatment effect of empagliflozin compared to placebo according to type 2 diabetes mellitus (T2DM) status. Hazard ratios (HR) and rate ratios (RR) based on Cox regression or negative binomial regression models adjusted for age, sex, estimated glomerular filtration rate (assessed categorically using the Chronic Kidney Disease Epidemiology Collaboration formula <45 vs. 45–<60 vs. 60–<90 vs. ≥90 ml/min/1.73 m^2^), geographical region, T2DM, persistent/permanent atrial fibrillation, prior myocardial infarction, peripheral artery disease, smoking status and left ventricular ejection fraction. Kaplan–Meier estimates and cumulative incidence function for the composite primary endpoint (*A*) and its components (*B*, *C*) and mean cumulative function for total number of heart failure hospitalizations (*D*). CI, confidence interval.

**Figure 5:**
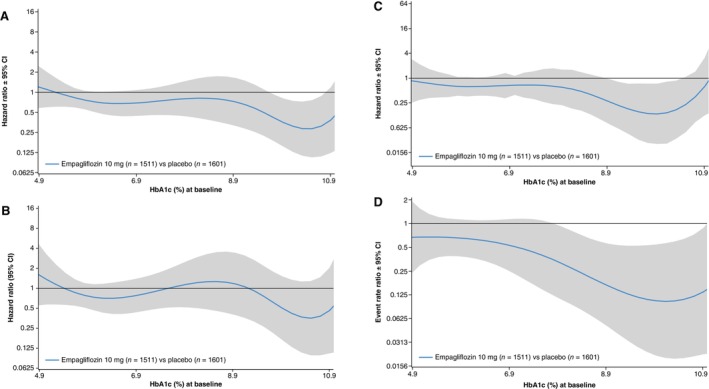
Treatment effect for empagliflozin versus placebo according to baseline glycated haemoglobin (HbA1c) as a continuous variable. (*A*) Time to first heart failure hospitalization or all‐cause mortality: empagliflozin versus placebo by baseline HbA1c. (*B*) Time to all‐cause mortality: empagliflozin versus placebo by baseline HbA1c. (*C*) Time to first heart failure hospitalization: empagliflozin versus placebo by baseline HbA1c. (*D*) Total number of heart failure hospitalizations: empagliflozin versus placebo by baseline HbA1c. The solid black line represents the hazard ratio of the treatment effect. The grey shaded area represents the 95% confidence interval (CI) around the treatment effects. The dotted horizontal line represents a hazard ratio of 1 (i.e. no difference between randomized groups). Hazard ratios and rate ratios based on Cox regression or negative binomial regression models adjusted for age, sex, estimated glomerular filtration rate (assessed categorically using the Chronic Kidney Disease Epidemiology Collaboration formula <45 vs. 45–<60 vs. 60–<90 vs. ≥90 ml/min/1.73 m^2^), geographical region, type 2 diabetes mellitus, persistent/permanent atrial fibrillation, prior myocardial infarction, peripheral artery disease, smoking status and left ventricular ejection fraction.

The effect of empagliflozin versus placebo on the primary endpoint was consistent in those with and without T2DM (*Figures* *3* and *4*). Empagliflozin reduced time to first HF hospitalization (T2DM: HR 0.85, 95% CI 0.58–1.23; no T2DM: HR 0.56, 95% CI 0.34–0.93; unknown HbA1c: HR 0.84, 95% CI 0.56–1.27, *p* for interaction 0.39) and total HF hospitalizations (T2DM: RR 0.62, 95% CI 0.39–0.98; no T2DM: RR 0.51, 95% CI 0.29–0.89; unknown HbA1c: RR 0.90, 95% CI 0.56–1.47, *p* for interaction 0.28) in patients with and without T2DM and in patients with unknown HbA1c. First adverse event of HF, total adverse events of HF, first and total adverse events of HF or all‐cause mortality were also reduced by empagliflozin to a similar extent in those with and without T2DM and patients with unknown HbA1c (online supplementary *Figure* *S2*). Empagliflozin reduced first and total HF hospitalizations across the range of baseline HbA1c (*Figure* *5*). The treatment effect of empagliflozin on the primary endpoint, its components and total hospitalizations according to T2DM status did not vary according to the prescription of metformin or insulin, or according to BMI, age, or sex at baseline (online supplementary *Figure* *S3*).

### Safety

The safety profile of empagliflozin and placebo was similar in participants with and without T2DM and those with unknown HbA1c (*Table* *2*). Specifically, there were no differences in rates of hypotension, volume depletion, and acute kidney injury between empagliflozin and placebo for those with T2DM and no T2DM and those with unknown HbA1c.

**Table 2:** Safety outcomes according to type 2 diabetes mellitus status^a^

	T2DM				No T2DM				Unknown HbA1c			
	Empagliflozin (*n* = 1069)		Placebo (*n* = 1070)		Empagliflozin (*n* = 902)		Placebo (*n* = 988)		Empagliflozin (*n* = 1263)		Placebo (*n* = 1171)	
	%	Incidence per 100 PY	%	Incidence per 100 PY	%	Incidence per 100 PY	%	Incidence per 100 PY	%	Incidence per 100 PY	*n* (%)	Incidence per 100 PY
Patients with any AE	28	26	30	29	30	29	30	30	26	23	23	20
Patients with AE leading to permanent treatment discontinuation	4	3	4	3	5	4	5	4	3	2	3	2
Patients with serious AE	24	22	27	25	25	23	27	27	23	19	21	18
Patients with AEs leading to LLA	0.7	0.5	0.4	0.3	0	0	0	0	0.2	0.1	0.1	0.1
Hepatic injury (AESI)^b^	0.1	0.1	0.1	0.1	0.3	0.3	0.1	0.1	0.3	0.2	0	0
Ketoacidosis (AESI)	0.2	0.2	0.1	0.1	0	0	0	0	0	0	0	0
Contrast‐induced AKI (AESI)	0.5	0.4	0.6	0.5	0	0	0.1	0.1	0.2	0.2	0.2	0.1
Acute kidney failure (narrow SMQ)^c^	2	1	2	2	1	1	2	1	1	0.8	2	1
AKI^d^	1	0.8	2	1	0.9	0.7	1	1	0.6	0.5	1	0.9
Volume depletion (narrow BIcMQ)	1	0.8	1	1	2	1	2	1	0.6	0.5	0.9	0.7
Hypotension (BIcMQ based)	1	0.8	1	0.9	2	1	1	1	0.6	0.5	0.9	0.7
Hypoglycaemia (narrow SMQ)^c^	0.4	0.3	0.5	0.4	0	0	0	0	0	0	0	0

AE, adverse event; AESI, adverse event of special interest; AKI, acute kidney injury; ALT, alanine aminotransferase; AST, aspartate aminotransferase; BIcMQ, Boehringer Ingelheim‐customized MedDRA query; HbA1c, glycated haemoglobin; LLA, lower limb amputation; MedDRA, Medical Dictionary for Regulatory Activities; PY, patient‐years; SMQ, standardized MedDRA query; T2DM, type 2 diabetes mellitus.

Events were identified with the use of a BIcMQ, version 26.1.

^a^ Patients who received at least one dose of empagliflozin or placebo were included in the safety population. Shown are adverse events analysed up to 7 days after the discontinuation of the trial regimen, except for LLA, which were analysed up to the end of the trial. AEs that were to be reported in the trial included serious AEs, AEs that led to discontinuation of the trial regimen for at least 7 days, and AESI, defined as ketoacidosis, AEs leading to LLA, hepatic injury, and contrast‐induced AKI.

^b^ Hepatic injury was defined as an AST level or an ALT level (or both) of at least three times the upper limit of the normal range, combined with a total bilirubin level of at least two times the upper limit of the normal range, as measured in the same blood sample or in blood samples obtained within 30 days of each other; or an ALT level or AST level (or both) of at least 10 times the upper limit of the normal range. Hepatic injury as defined by these criteria and reported by the investigator occurred in four patients in the empagliflozin group and in no patients in the placebo group.

^c^ Events were identified with the use of a SMQ, version 26.1.

^d^ ‘Acute kidney injury’ is a MedDRA, version 26.1, preferred term.

## Discussion

In the EMPACT‐MI trial, T2DM was common and rates of the primary outcome and mortality (all‐cause and cardiovascular) were higher in patients with than without T2DM. In contrast, rates for HF hospitalizations (first or total) were not significantly higher in those with T2DM. In patients with and without T2DM, empagliflozin substantially reduced first and total HF hospitalizations as well as HF adverse events (*Graphical Abstract*).

That T2DM is common in populations with acute MI is well established. The prevalence of T2DM in all‐comer acute MI cohorts has previously been reported to be between 15% and 25%.^1^, ^2^, ^11^ The proportion of participants with T2DM in EMPACT‐MI (32%) was similar to that reported in prior acute MI trials in populations at high risk of HF (low LVEF ± congestion). For example, in VALIANT, EPHESUS and PARADISE‐MI 23%, 32% and 42%, respectively, had T2DM at baseline.^12^, ^13^, ^14^ As T2DM was an enrichment HF criterion in EMPACT‐MI, the proportion of patients with T2DM is likely to be higher than in real‐world populations. Remarkably, only 16% of the EMPACT‐MI had confirmed normal glycaemic status. Glycaemic status in acute MI has not been widely investigated in randomized trials. In a single centre cohort of 841 patients, 17% had normal glycaemic status.^15^ A major finding of interest in EMPACT‐MI was the large number of patients (more than one in three) where there was no HbA1c available to ascertain T2DM status. The European Society of Cardiology (ESC) guidelines for management of acute MI recommend assessment of glycaemic status in all patients during acute MI (ESC class IB recommendation).^16^ Using the opportunity of an acute coronary presentation to establish T2DM status allows appropriate glycaemic control as well as prescription of therapies that are indicated for chronic management of T2DM. Few patients in EMPACT‐MI (1.3%) were found to have previously undiagnosed T2DM. How many of those who did not have an HbA1c measured had T2DM which remained undiagnosed is unknown. Studies investigating the impact of screening strategies in acute coronary syndromes have yet to be performed.

Rates of cardiovascular events after acute MI have long been known to be higher in those with T2DM than those without.^1^, ^2^ In EMPACT‐MI the higher event rates with T2DM was striking for mortality. All‐cause and cardiovascular mortality were 70% and 132% higher, respectively, in those with T2DM when compared to those without T2DM. The increased mortality risk conferred by T2DM in EMPACT‐MI is greater than seen in previous similar trials. For example, in the VALIANT trial which enrolled patients from 1998–2001, T2DM was associated with a 37% and 42% increased risk of all‐cause and cardiovascular mortality, respectively.^2^ EMPACT‐MI was a streamlined trial which relied on trained investigators to report and classify events. Investigations into causes of this high mortality rate in those with T2DM in the current era are warranted. Understanding why T2DM is associated with higher mortality would allow for development of targeted therapies. Potential causes of higher death rates in T2DM following acute MI include more stent thromboses, in‐stent restenoses, myocardial rupture, fatal dysrhythmias, bleeding or deaths due to HF. The absence of a clear higher HF hospitalization event rate in those with T2DM after adjustment in EMPACT‐MI is surprising. In previous acute MI trials, T2DM has been associated with higher rates of HF hospitalizations.^2^, ^17^, ^18^ In EMPACT‐MI there was a numerical but not statistically significant higher rate of HF hospitalizations in those with T2DM after comprehensive multivariable adjustment. Why the risk conferred by T2DM might be less than previously seen might be related to changing drivers of HF hospitalization after acute MI. Modern rapid and complete revascularization, optimal acute MI medical therapy and improved comprehensive post‐MI management may have reduced the excess risk of HF hospitalization conferred by T2DM. It is unlikely that use of glucose‐lowering therapies is responsible as, except for SGLT2 inhibitors, these agents have not been reported to reduce HF hospitalizations.

The consistency of the treatment effect of empagliflozin on HF hospitalizations in those with and without T2DM following acute MI is similar to the effects of SGLT2 inhibitors in other disease states. In HF with reduced (HFrEF) and preserved ejection fraction (HFpEF), empagliflozin and dapagliflozin have shown very similar reductions in HF events in those with and without T2DM. Consistency of SGLT2 inhibitor treatment effects in HFrEF and HFpEF populations are also seen across the spectrum of baseline HbA1c. The treatment effect of SGLT2 inhibitors on reducing HF hospitalizations following acute MI in EMPACT‐MI confirms the benefit in both those with and without T2DM. EMPACT‐MI was the first SGLT2 inhibitor trial in acute MI to enrol those without T2DM as well as being the only post‐MI SGLT2 inhibitor trial to investigate effects on clinical outcomes. DAPA‐MI only included those without T2DM and had a primary endpoint of seven ‘cardio‐metabolic’ components analysed by the win ratio.^7^ In the only completed mechanistic trial of SGLT2 inhibitors after MI, the EMMY trial, those with and without T2DM were enrolled. Empagliflozin reduced N‐terminal pro‐B‐type natriuretic peptide and left ventricular volumes and increased LVEF.^19^ A second acute MI mechanistic trial, EMPRESS‐MI (NCT05020704), which enrolled patients with and without T2DM will complete shortly. The effect of empagliflozin on cardiac magnetic resonance imaging‐measured left ventricular volumes, renal parameters and circulating biomarkers will be reported. In trials of SGLT2 inhibitors in chronic kidney disease, HF benefits were seen regardless of the presence or absence of T2DM.^20^ The consistency of reduction in HF hospitalizations across different clinical conditions regardless of presence or absence of T2DM (as well as across the spectrum of baseline HbA1c) gives a clear indication that the mechanism of reduction in HF with SGLT2 inhibitors is independent of any glucose‐related or diabetes‐related factors. Several mechanistic trials in HF have indicated a variety of different glucose‐ and diabetes‐independent mechanisms of HF benefit.

Empagliflozin did not reduce all‐cause mortality following MI whether T2DM was present or absent. This lack of treatment effect suggests that the causes of death in EMPACT‐MI are not modifiable by SGLT2 inhibitors regardless of diabetes status. The very high rates of death in those with T2DM highlights an ongoing and unmet need. Alternative, novel treatment strategies to further improve outcomes following MI, especially in patients with T2DM, are necessary.^21^ Potential causes of death that might not be impacted by SGLT2 inhibitors include arrhythmias (tachycardia and bradycardia events), stent thrombosis, in‐stent restenosis, myocardial rupture, pulmonary emboli, and other cardiovascular and non‐cardiovascular causes.

The safety of empagliflozin was similar in those with and without T2DM. In the context of acute MI with recent percutaneous intervention, haemodynamic fluctuations, and initiation of multiple pharmacological therapies, there was no increase in adverse events compared to placebo.

The major limitation of the current analysis is the lack of systematic measurement of HbA1c in EMPACT‐MI. The lack of systematic HbA1c measurement prevented precise ascertainment of glycaemia status in all patients. The lack of systematic measurement of HbA1c also meant that our analysis of treatment effect of empagliflozin across baseline HbA1c is subject to indication bias. The current analysis did not allow a meaningful analysis of pre‐diabetes as a standalone subgroup. Of the 447 patients with pre‐diabetes, only 39 had a primary outcome event (online supplementary *Figure* *S4*). The other limitations of EMPACT‐MI are the low numbers of women and low numbers of racial and ethnic minorities.

In conclusion, around one third of patients in EMPACT‐MI had T2DM and only 16% were normoglycaemic. Patients with T2DM were at much higher risk of mortality than those without T2DM. Empagliflozin reduced first and total HF hospitalizations, but not all‐cause mortality, regardless of the presence or absence of T2DM or unknown HbA1c.

### Funding

The manuscript was sponsored by the Boehringer Ingelheim and Eli Lilly and Company Diabetes Alliance. Graphical assistance was provided by HCG which was contracted, and compensated by Boehringer Ingelheim.

**Conflict of interest**: M.C.P. reports research funding: Boehringer Ingelheim, Roche, SQ Innovations, AstraZeneca, Novartis, Novo Nordisk, Medtronic, Boston Scientific, Pharmacosmos; consultancy and trial committees: Akero, Applied Therapeutics, Amgen, AnaCardio, Biosensors, Boehringer Ingelheim, Novartis, AstraZeneca, Novo Nordisk, Abbvie, Bayer, Horizon Therapeutics, Takeda, Cardiorentis, Pharmacosmos, Siemens, Eli Lilly, Vifor, New Amsterdam, Moderna, Teikoku, LIB Therapeutics, 3R Lifesciences. J.A.U. reports advisory board: Boehringer Ingelheim, Novavax, Novo Nordisk, Sanofi; Speaker honoraria: Amgen, AstraZeneca, Boehringer Ingelheim, Eli Lilly and Company; research funding to his institution: Amgen, Bayer, Boehringer Ingelheim, Novartis. S.D.A. has received research support from Abbott Vascular and Vifor International, and personal fees from Boehringer Ingelheim, Bayer, AstraZeneca, Novartis, Vifor International, Impulse Dynamics, Respicardia, and St Jude Medical. W.S.J. reports research grants from Bayer, Boehringer Ingelheim, Merck, Novartis, PCORI, NIH. P.v.d.M. is supported by a grant from the European Research Council (ERC CoG 101 045 236, DISSECT‐HF). The UMCG, which employs P.v.d.M., received consultancy fees and/or grants from Novartis, Pharmacosmos, Vifor Pharma, AstraZeneca, Pfizer, Pharma Nord, BridgeBio, Novo Nordisk, Daiichi Sankyo, Boehringer Ingelheim and Ionis. O.A. reports national PI‐steering committee member for the study and participated in paid lectures and advisory board meetings and have clinical trials in his department for Boehringer Ingelheim. M.C.B. reports modest honorarium from MSD, Pfizer, Bristol‐Myers Squibb, CSL Behring, Janssen, Boehringer Ingelheim, Anthos Therapeutics. J.B. received honoraria for lectures/consulting from Novartis, Vifor, Bayer, Pfizer, Boehringer Ingelheim, AstraZeneca, Cardior, CVRx, BMS, Amgen, Corvia, Norgine, Edwards, Roche not related to this article; and research support for the department from Zoll, CVRx, Abiomed, Norgine, Roche, not related to this article. A.B.G. has lectured and/or participated in advisory boards for Abbott, AstraZeneca, Bayer, Boehringer Ingelheim, Medtronic, Novartis, Novo Nordisk, Roche Diagnostics, Vifor. V.K.C. reports speaking fee from Novartis, Sanofi, Astra, Boehringer, Novo Nordisk, Pfizer, Sun Pharma, Mankind, Lupin. J.L.J. reports a board position with Imbria Pharma, grant support from Abbott, Applied Therapeutics, AstraZeneca, BMS, Novartis Pharmaceuticals, consulting income from Abbott Diagnostics, Beckman‐Coulter, BMS, Boehringer Ingelheim, Jana Care, Janssen, Lilly, Novartis, Prevencio, Quidel, and Roche Diagnostics, and serves on clinical endpoint committees/data safety monitoring boards for Abbott, AbbVie, Amgen, CVRx, Medtronic, Pfizer, and Roche Diagnostics. R.D.L. reports research grants or contracts from Amgen, Bristol‐Myers Squibb, GlaxoSmithKline, Medtronic, Pfizer, Sanofi‐Aventis; funding for educational activities or lectures from Pfizer, Daiichi Sankyo, and Novo Nordisk; and funding for consulting or other services from Bayer, Boehringer Ingelheim, Bristol‐Myers Squibb, Novo Nordisk. P.P. reports personal fees from Boehringer Ingelheim, AstraZeneca, Servier, Bristol Myers Squibb, Amgen, Novartis, Merck, Pfizer, Berlin Chemie, and grants and personal fees from Vifor Pharma. M.S. reports lecture fees from Novartis, AstraZeneca, Bohringer and Novo Nordisk. S.Z. reports research grant support, served on advisory boards for, or speaker engagements with AstraZeneca, Bayer, BMS, Boehringer Ingelheim, Cytokinetics, Eli Lilly, GSK, Janssen, Medtronic, Merck, Novartis, Novo‐Nordisk, Otsuka, Pfizer, Roche, Salubrisbio, Servier and Vifor Pharma; and serves on a clinical trial committee or as a national lead for studies sponsored by AstraZeneca, Bayer, Boehringer Ingelheim, Merck, Novartis and Pfizer; non industry: Canadian Medical and Surgical KT Group, CCS, CHFS, Charite, EOCI, Liv, Medscape, Ology, PACE‐CME, Radcliffe, Reach MD, Translational Medicine Academy. D.L.B. reports advisory board: Angiowave, Bayer, Boehringer Ingelheim, CellProthera, Cereno Scientific, Elsevier Practice Update Cardiology, High Enroll, Janssen, Level Ex, McKinsey, Medscape Cardiology, Merck, MyoKardia, NirvaMed, Novo Nordisk, PhaseBio, PLx Pharma, Stasys; Board of Directors: American Heart Association New York City, Angiowave (stock options), Bristol Myers Squibb (stock), DRS.LINQ (stock options), High Enroll (stock); consultant: Broadview Ventures, GlaxoSmithKline, Hims, SFJ, Youngene; Data Monitoring Committees: Acesion Pharma, Assistance Publique‐Hôpitaux de Paris, Baim Institute for Clinical Research (formerly Harvard Clinical Research Institute, for the PORTICO trial, funded by St. Jude Medical, now Abbott), Boston Scientific (Chair, PEITHO trial), Cleveland Clinic, Contego Medical (Chair, PERFORMANCE 2), Duke Clinical Research Institute, Mayo Clinic, Mount Sinai School of Medicine (for the ENVISAGE trial, funded by Daiichi Sankyo; for the ABILITY‐DM trial, funded by Concept Medical; for ALLAY‐HF, funded by Alleviant Medical), Novartis, Population Health Research Institute; Rutgers University (for the NIH‐funded MINT Trial); Honoraria: American College of Cardiology (Senior Associate Editor, Clinical Trials and News, ACC.org; Chair, ACC Accreditation Oversight Committee), Arnold and Porter law firm (work related to Sanofi/Bristol‐Myers Squibb clopidogrel litigation), Baim Institute for Clinical Research (formerly Harvard Clinical Research Institute; RE‐DUAL PCI clinical trial steering committee funded by Boehringer Ingelheim; AEGIS‐II executive committee funded by CSL Behring), Belvoir Publications (Editor in Chief, Harvard Heart Letter), Canadian Medical and Surgical Knowledge Translation Research Group (clinical trial steering committees), CSL Behring (AHA lecture), Cowen and Company, Duke Clinical Research Institute (clinical trial steering committees, including for the PRONOUNCE trial, funded by Ferring Pharmaceuticals), HMP Global (Editor in Chief, Journal of Invasive Cardiology), Journal of the American College of Cardiology (Guest Editor; Associate Editor), K2P (Co‐Chair, interdisciplinary curriculum), Level Ex, Medtelligence/ReachMD (CME steering committees), MJH Life Sciences, Oakstone CME (Course Director, Comprehensive Review of Interventional Cardiology), Piper Sandler, Population Health Research Institute (for the COMPASS operations committee, publications committee, steering committee, and USA national co‐leader, funded by Bayer), WebMD (CME steering committees), Wiley (steering committee); other: Clinical Cardiology (Deputy Editor); Patent: Sotagliflozin (named on a patent for sotagliflozin assigned to Brigham and Women's Hospital who assigned to Lexicon; neither I nor Brigham and Women's Hospital receive any income from this patent); research funding: Abbott, Acesion Pharma, Afimmune, Aker Biomarine, Alnylam, Amarin, Amgen, AstraZeneca, Bayer, Beren, Boehringer Ingelheim, Boston Scientific, Bristol‐Myers Squibb, Cardax, CellProthera, Cereno Scientific, Chiesi, CinCor, Cleerly, CSL Behring, Eisai, Ethicon, Faraday Pharmaceuticals, Ferring Pharmaceuticals, Forest Laboratories, Fractyl, Garmin, HLS Therapeutics, Idorsia, Ironwood, Ischemix, Janssen, Javelin, Lexicon, Lilly, Medtronic, Merck, Moderna, MyoKardia, NirvaMed, Novartis, Novo Nordisk, Otsuka, Owkin, Pfizer, PhaseBio, PLx Pharma, Recardio, Regeneron, Reid Hoffman Foundation, Roche, Sanofi, Stasys, Synaptic, The Medicines Company, Youngene, 89Bio; royalties: Elsevier (Editor, Braunwald's Heart Disease); site co‐investigator: Abbott, Biotronik, Boston Scientific, CSI, Endotronix, St. Jude Medical (now Abbott), Philips, SpectraWAVE, Svelte, Vascular Solutions; Trustee: American College of Cardiology; Unfunded Research: FlowCo. A.F.H. reports consultant to Amgen, AstraZeneca, Bayer, Boehringer Ingelheim, Boston Scientific, Bristol Myers Squibb, Cytokinetics, Eidos, GlaxoSmithKline, Intellia, Intercept, Myokardia, Novartis, Novo Nordisk, Prolaio, TikkunLev; research funding: American Regent, Amgen, Bayer, Boehringer Ingelheim, Lilly, Merck, Novartis, Novo Nordisk and Verily. J.B. reports consultant to Abbott, American Regent, Amgen, Applied Therapeutic, AskBio, Astellas, AstraZeneca, Bayer, Boehringer Ingelheim, Boston Scientific, Bristol Myers Squibb, Cardiac Dimension, Cardiocell, Cardior, CSL Bearing, CVRx, Cytokinetics, Daxor, Edwards, Element Science, Faraday, Foundry, G3P, Innolife, Impulse Dynamics, Imbria, Inventiva, Ionis, Lexicon, Lilly, LivaNova, Janssen, Medtronics, Merck, Occlutech, Owkin, Novartis, Novo Nordisk, Pfizer, Pharmacosmos, Pharmain, Prolaio, Regeneron, Renibus, Roche, Salamandra, Sanofi, SC Pharma, Secretome, Sequana, SQ Innovation, Tenex, Tricog, Ultromics, Vifor, and Zoll. M.M., T.G., M.B. and M.S. are employees of Boehringer Ingelheim. All other authors have nothing to disclose.

## Supporting information


**Supplementary Figure S1.** Outcomes according to baseline T2DM status in the placebo group.
Hazard ratios and Rate Ratios based on Cox regression or Negative binomial regression models adjusted for age, sex, estimated glomerular filtration rate (assessed categorically using the CKD‐EPI formula <45 vs 45–<60 vs 60–<90 vs ≥90 mL/min/1.73 m^2^), geographical region, type 2 diabetes, persistent/permanent atrial fibrillation, prior MI, peripheral artery disease, smoking status and LVEF. Kaplan–Meier Estimates and Cumulative Incidence Function for the Composite Primary End Point and Its Components and Mean cumulative function for total number of heart failure hospitalizations.

**Supplementary Figure S2.** Treatment effect of empagliflozin compared to placebo on HF AEs according to T2DM status. Hazard ratios and Rate Ratios based on Cox regression or Negative binomial regression models adjusted for age, sex, estimated glomerular filtration rate (assessed categorically using the CKD‐EPI formula <45 vs 45–<60 vs 60–<90 vs ≥90 mL/min/1.73 m^2^), geographical region, type 2 diabetes, persistent/permanent atrial fibrillation, prior MI, peripheral artery disease, smoking status and LVEF. Kaplan–Meier Estimates and Cumulative Incidence Function for the Composite Primary End Point and Its Components and Mean cumulative function for total number of heart failure hospitalizations.

**Supplementary Figure S3.** (A and B) Treatment effect for empagliflozin versus placebo according to baseline metformin or insulin prescription, BMI, age and sex. Hazard ratios and based on Cox regression or Negative binomial regression models adjusted for age (if not part of subgroup), sex (if not part of subgroup), estimated glomerular filtration rate (assessed categorically using the CKD‐EPI formula <45 vs 45–<60 vs 60–<90 vs ≥90 mL/min/1.73 m^2^), geographical region, subgroup, persistent/permanent atrial fibrillation, prior MI, peripheral artery disease, smoking status and LVEF (categorical or continuous), treatment and interaction of subgroup and treatment. T2DM is defined as diagnosed T2DM (investigator‐reported) and undiagnosed T2D (i.e. baseline HbA1c > =6.5%). n number of patients with event (for time to first event endpoints) or number of events (for total number of events endpoint) based on N number of patients at risk. No T2DM is defined as normoglycaemia or pre‐diabetes and unknown diabetes status (i.e. no T2DM without HbA1c measured).

**Supplementary Figure S4.** Effect of empagliflozin according to baseline glycaemic status (including prediabetes). Hazard ratios and Rate Ratios based on Cox regression or Negative binomial regression models adjusted for age, sex, estimated glomerular filtration rate (assessed categorically using the CKD‐EPI formula <45 vs 45–<60 vs 60–<90 vs ≥90 mL/min/1.73 m^2^), geographical region, type 2 diabetes, persistent/permanent atrial fibrillation, prior MI, peripheral artery disease, smoking status and LVEF (categorical or continuous). T2DM is defined as diagnosed T2DM (investigator‐reported) and undiagnosed T2D (i.e. baseline HbA1c > =6.5%). n number of patients with event (for time to first event endpoints) or number of events (for total number of events endpoint) based on N number of patients at risk. Pre‐diabetes is defined as HbA1c ≥5.7% (≥39 mmol/mL) and <6.5% (<48 mmol/mol) measured during the index presentation excluding those with baseline diagnosed T2DM. Unknown refers to no T2DM without HbA1c measured.

**Supplementary Table S1.** Baseline characteristics according T2DM status, and treatment assignment to empagliflozin or placebo.
**Supplementary Table S2.** Diabetes therapies at baseline and at discharge according to T2DM status.
