# Supplementary material for: Empagliflozin in acute myocardial infarction in patients with and without type 2 diabetes: A pre‐specified analysis of the EMPACT‐MI trial
Source: Eur J Heart Fail. 2024 Dec 26;27(3):577–88. doi: 10.1002/ejhf.3548 (PMC11955319; doi:10.1002/ejhf.3548)
Supplement: Supplementary file 1 — Supplementary Figure S1. Outcomes according to baseline T2DM status in the placebo group. Hazard ratios and Rate Ratios based on Cox regression or Negative binomial regression models adjusted for age, sex, estimated glomerular filtration rate (assessed categorically using the CKD‐EPI formula <45 vs 45–<60 vs 60–<90 vs ≥90 mL/min/1.73 m2), geographical region, type 2 diabetes, persistent/permanent atrial fibrillation, prior MI, peripheral artery disease, smoking status and LVEF. Kaplan–Meier Estimates and Cumulative Incidence Function for the Composite Primary End Point and Its Components and Mean cumulative function for total number of heart failure hospitalizations. [file EJHF-27-577-s004.pdf]

A. Primary endpoint (first heart failure hospitalization or all-cause mortality)

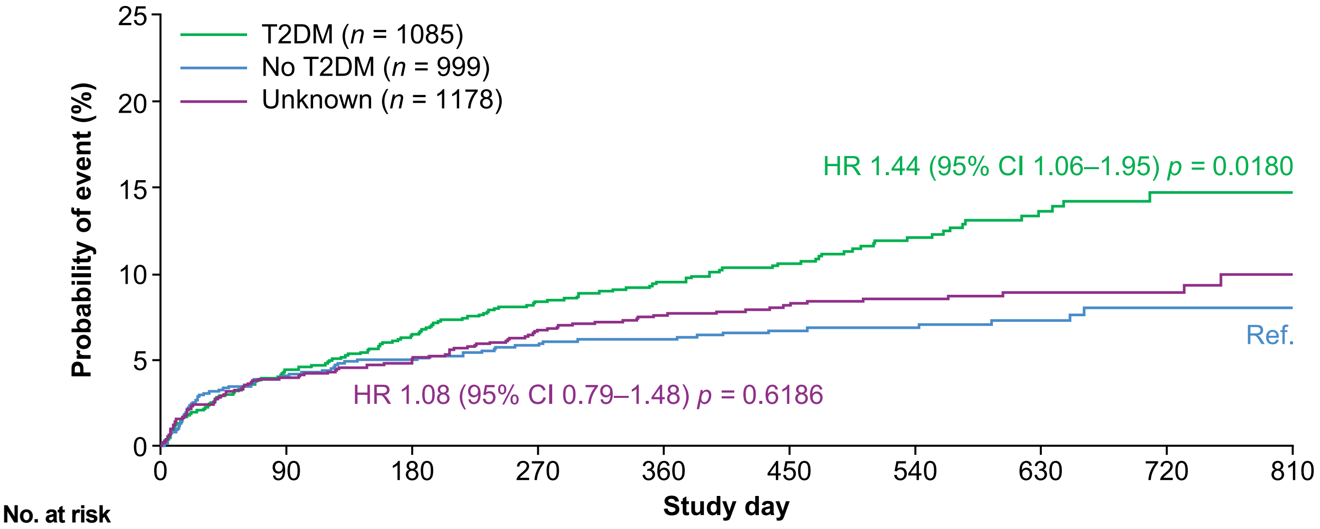

No. at risk										
T2DM	1085	1024	1002	932	815	675	500	310	160	51
No T2DM	999	942	927	871	759	637	473	305	144	25
Unknown	1178	1126	1115	1029	912	759	583	425	247	61

B. All-cause mortality

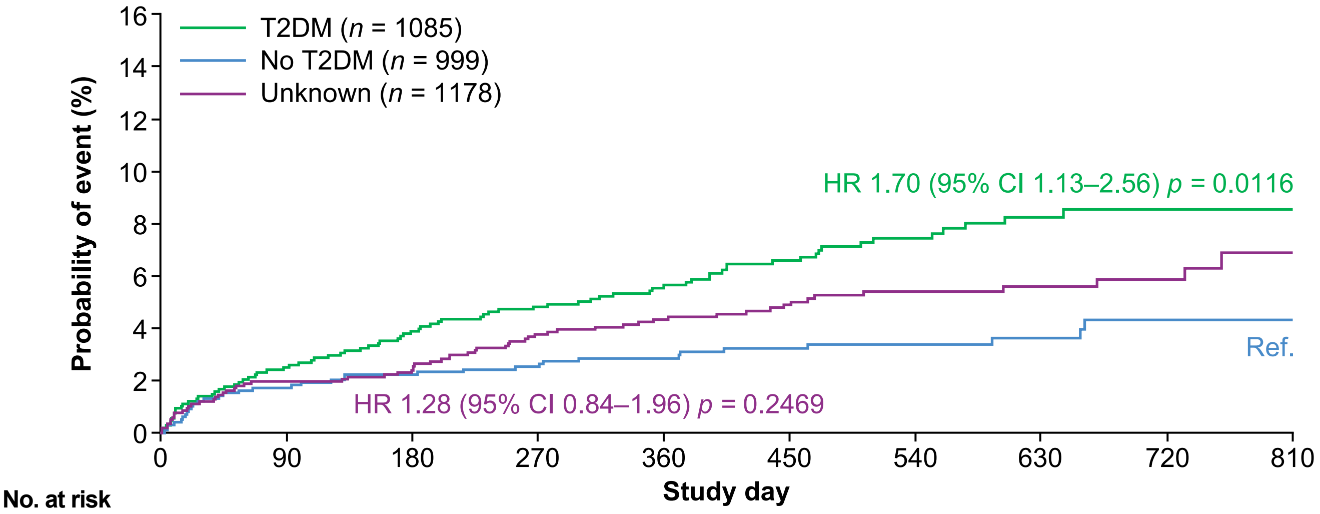

No. at risk										
T2DM	1085	1057	1040	983	865	721	538	338	178	54
No T2DM	999	975	969	917	807	683	507	327	157	29
Unknown	1178	1154	1150	1075	960	803	615	446	258	65

C. First heart failure hospitalization

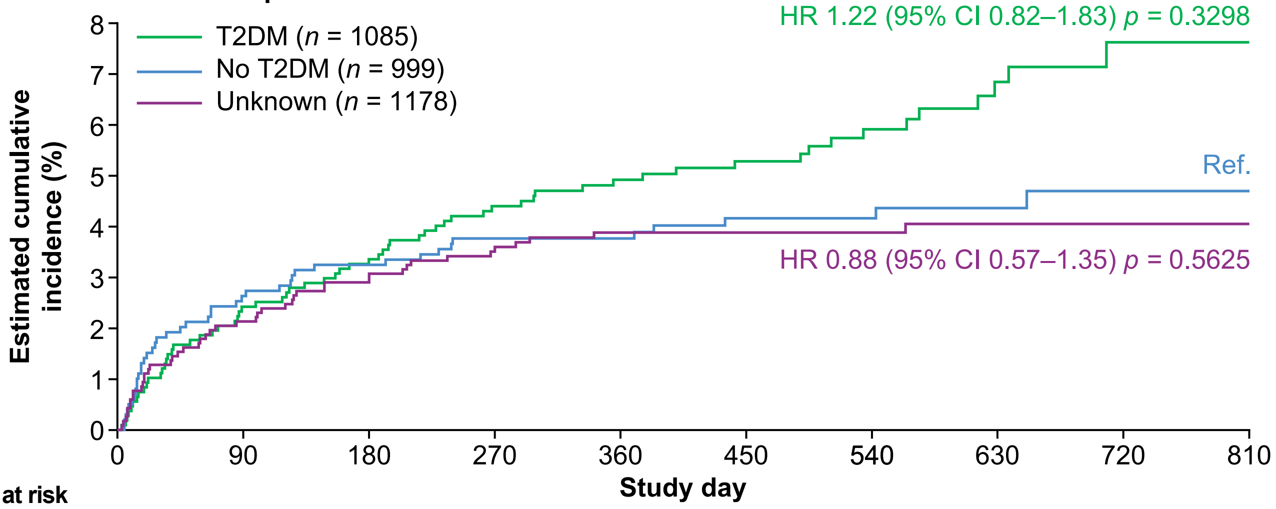

No. at risk										
T2DM	1085	1024	1002	932	815	675	500	310	160	51
No T2DM	999	942	927	871	759	637	473	305	144	25
Unknown	1178	1126	1115	1029	912	759	583	425	247	61

D. Total number of heart failure hospitalizations

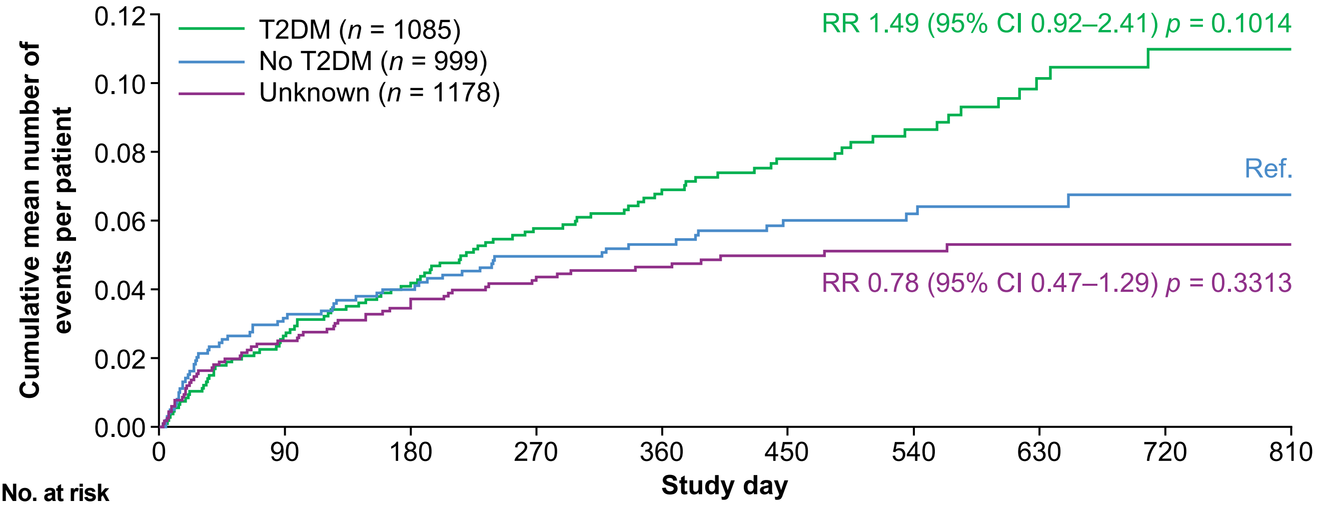

No. at risk										
T2DM	1085	1044	1027	963	845	701	521	326	173	53
No T2DM	999	965	954	902	790	666	493	319	152	28
Unknown	1178	1149	1144	1060	944	787	603	441	256	63
