# Supplementary material for: Empagliflozin in acute myocardial infarction in patients with and without type 2 diabetes: A pre‐specified analysis of the EMPACT‐MI trial
Source: Eur J Heart Fail. 2024 Dec 26;27(3):577–88. doi: 10.1002/ejhf.3548 (PMC11955319; doi:10.1002/ejhf.3548)

A. Time to first AE of HF by diabetes status (*p*-value for interaction = 0.2695)

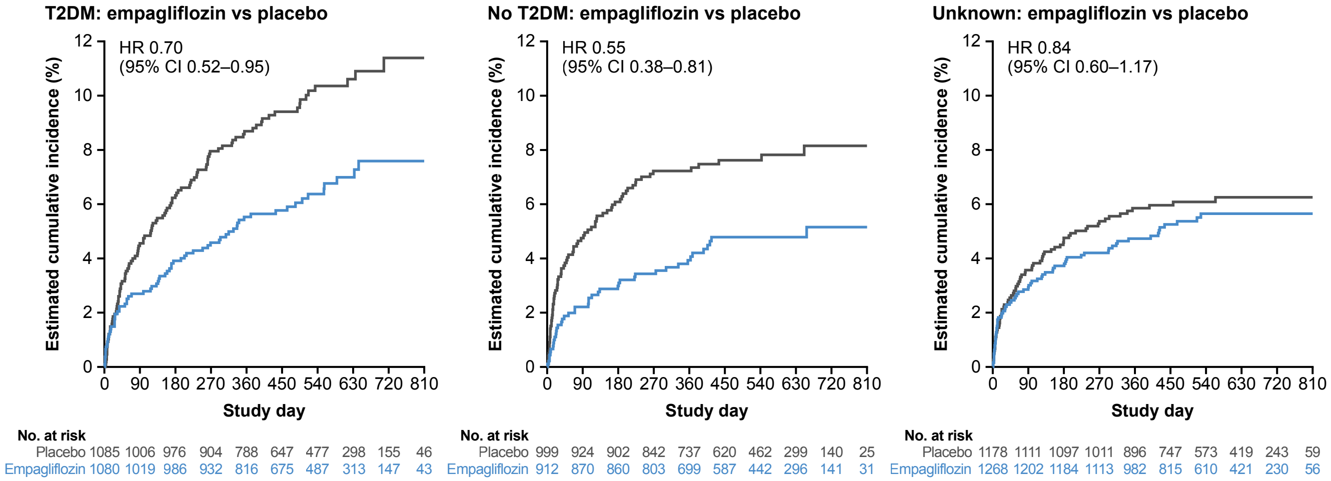

B. Total number of AEs of HF or all-cause mortality by diabetes status (*p*-value for interaction = 0.1327)

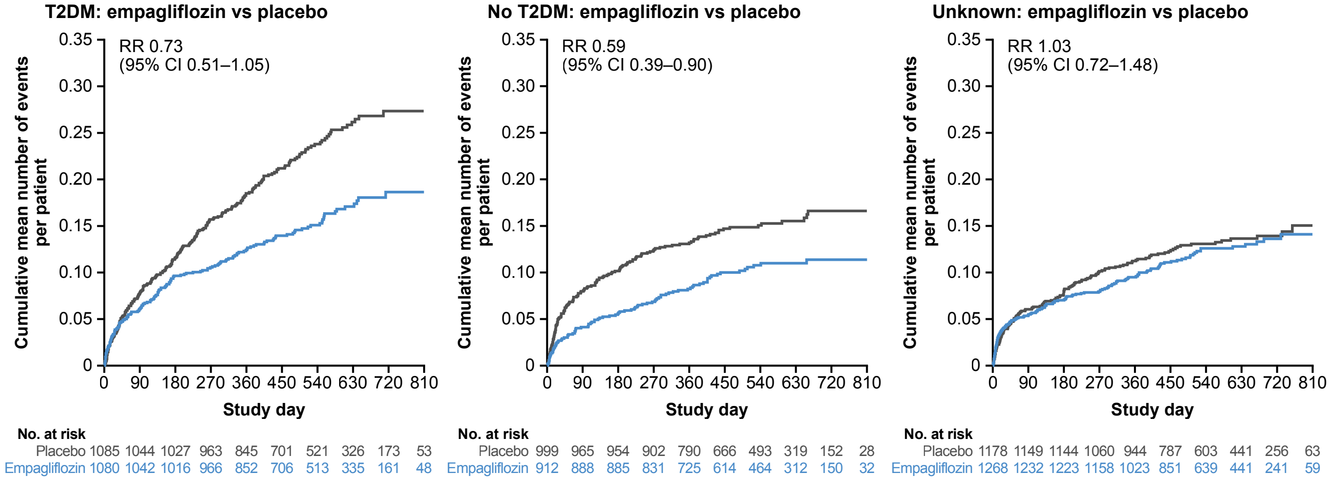

C. Time to first AE of HF or all-cause mortality by diabetes status (*p*-value for interaction = 0.4633)

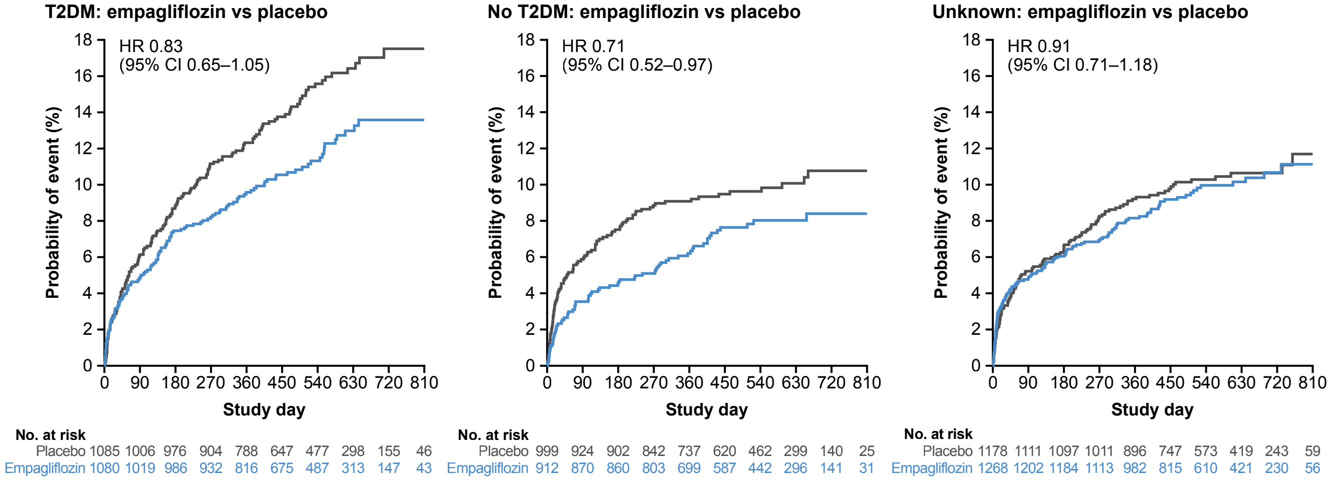

Supplement: Supplementary file 2 — Supplementary Figure S2. Treatment effect of empagliflozin compared to placebo on HF AEs according to T2DM status. Hazard ratios and Rate Ratios based on Cox regression or Negative binomial regression models adjusted for age, sex, estimated glomerular filtration rate (assessed categorically using the CKD‐EPI formula <45 vs 45–<60 vs 60–<90 vs ≥90 mL/min/1.73 m2), geographical region, type 2 diabetes, persistent/permanent atrial fibrillation, prior MI, peripheral artery disease, smoking status and LVEF. Kaplan–Meier Estimates and Cumulative Incidence Function for the Composite Primary End Point and Its Components and Mean cumulative function for total number of heart failure hospitalizations. [file EJHF-27-577-s005.pdf]
