# Supplementary figures and images for: Empagliflozin in acute myocardial infarction in patients with and without type 2 diabetes: A pre‐specified analysis of the EMPACT‐MI trial
Source: Eur J Heart Fail. 2024 Dec 26;27(3):577–88. doi: 10.1002/ejhf.3548 (PMC11955319; doi:10.1002/ejhf.3548)

A - Time to first heart failure hospitalization or all-cause mortality

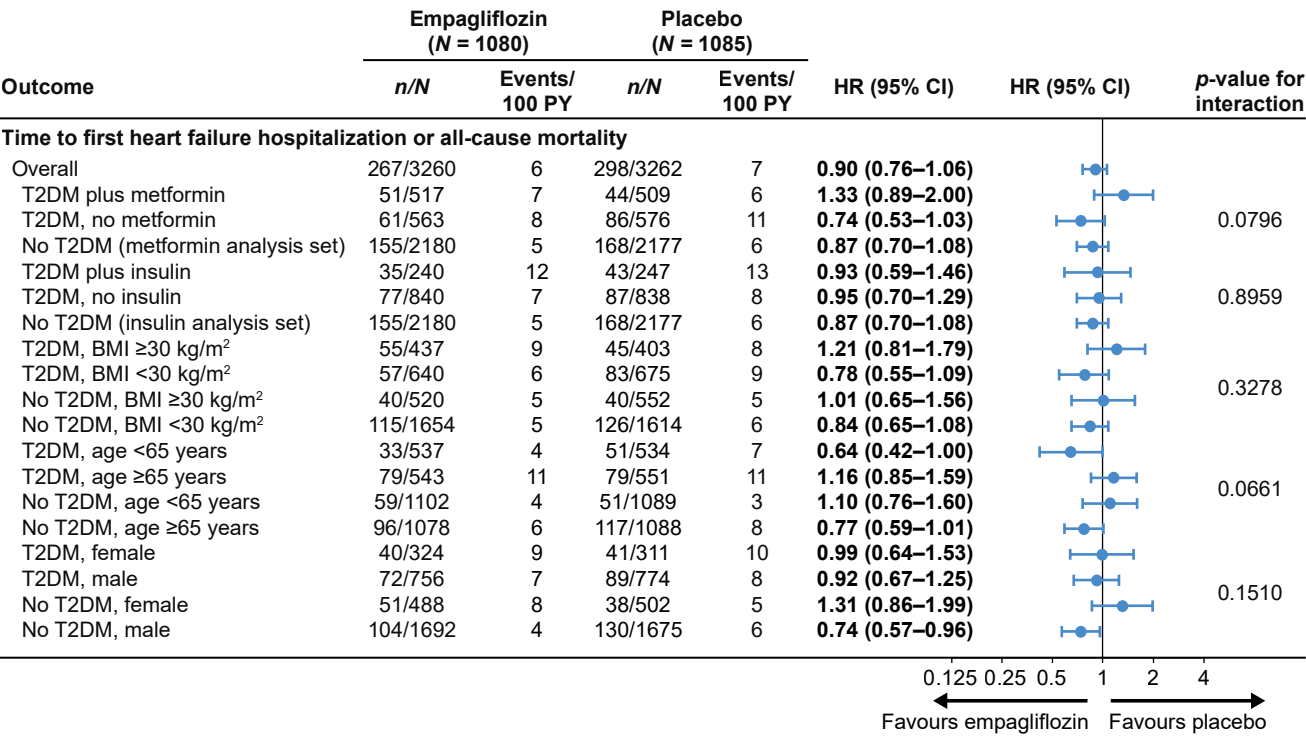

B - All-cause mortality

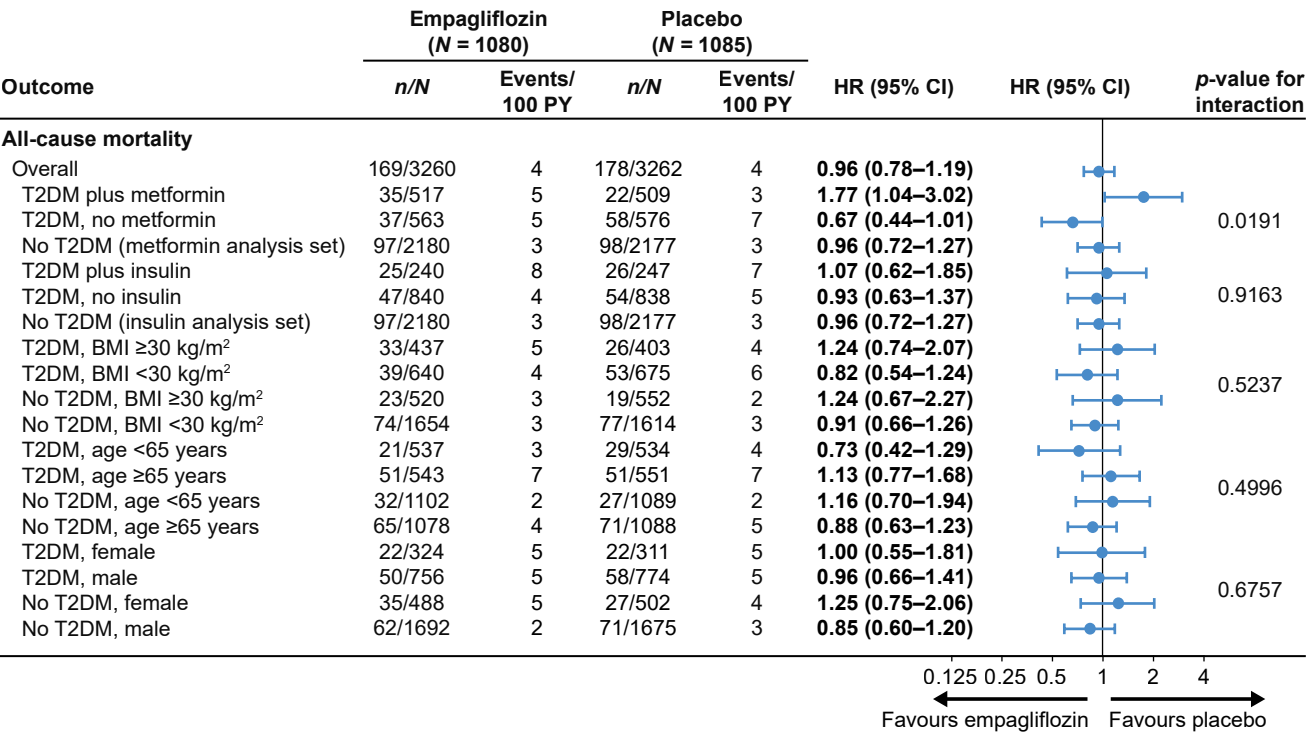

Supplement: Supplementary file 3 — Supplementary Figure S3. (A and B) Treatment effect for empagliflozin versus placebo according to baseline metformin or insulin prescription, BMI, age and sex. Hazard ratios and based on Cox regression or Negative binomial regression models adjusted for age (if not part of subgroup), sex (if not part of subgroup), estimated glomerular filtration rate (assessed categorically using the CKD‐EPI formula <45 vs 45–<60 vs 60–<90 vs ≥90 mL/min/1.73 m2), geographical region, subgroup, persistent/permanent atrial fibrillation, prior MI, peripheral artery disease, smoking status and LVEF (categorical or continuous), treatment and interaction of subgroup and treatment. T2DM is defined as diagnosed T2DM (investigator‐reported) and undiagnosed T2D (i.e. baseline HbA1c > =6.5%). n number of patients with event (for time to first event endpoints) or number of events (for total number of events endpoint) based on N number of patients at risk. No T2DM is defined as normoglycaemia or pre‐diabetes and unknown diabetes status (i.e. no T2DM without HbA1c measured). [file EJHF-27-577-s003.zip › Suppl Fig 3A.pdf]

C - Time to first heart failure hospitalization

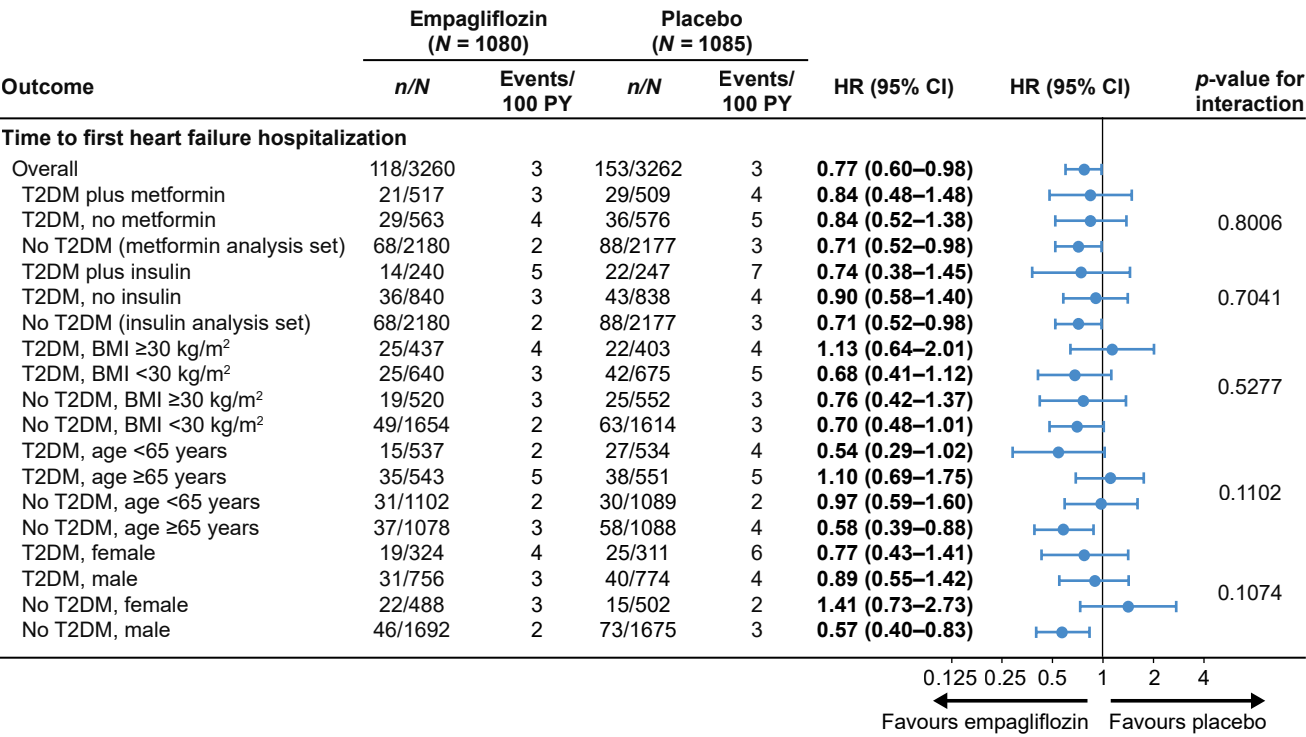

D - Total heart failure hospitalization

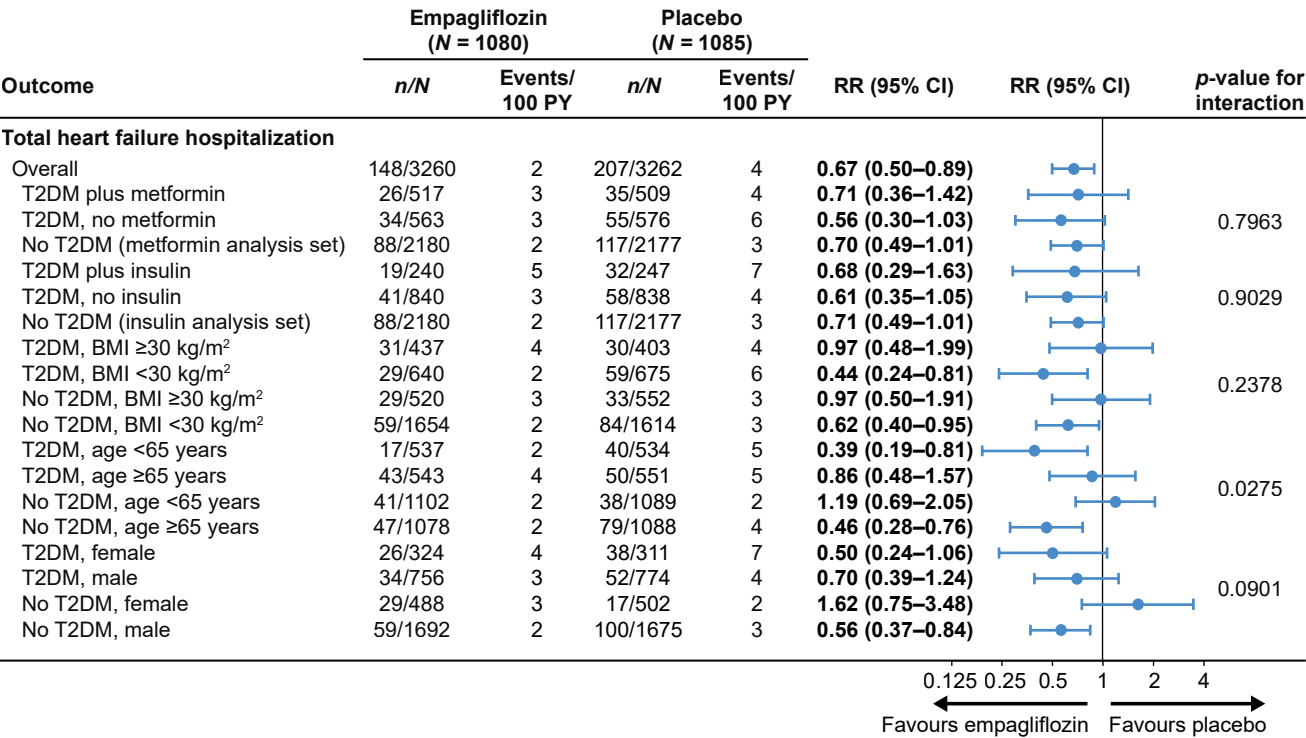

Supplement: Supplementary file 3 — Supplementary Figure S3. (A and B) Treatment effect for empagliflozin versus placebo according to baseline metformin or insulin prescription, BMI, age and sex. Hazard ratios and based on Cox regression or Negative binomial regression models adjusted for age (if not part of subgroup), sex (if not part of subgroup), estimated glomerular filtration rate (assessed categorically using the CKD‐EPI formula <45 vs 45–<60 vs 60–<90 vs ≥90 mL/min/1.73 m2), geographical region, subgroup, persistent/permanent atrial fibrillation, prior MI, peripheral artery disease, smoking status and LVEF (categorical or continuous), treatment and interaction of subgroup and treatment. T2DM is defined as diagnosed T2DM (investigator‐reported) and undiagnosed T2D (i.e. baseline HbA1c > =6.5%). n number of patients with event (for time to first event endpoints) or number of events (for total number of events endpoint) based on N number of patients at risk. No T2DM is defined as normoglycaemia or pre‐diabetes and unknown diabetes status (i.e. no T2DM without HbA1c measured). [file EJHF-27-577-s003.zip › Suppl Fig 3B.pdf]
