# Supplementary material for: Empagliflozin in acute myocardial infarction in patients with and without type 2 diabetes: A pre‐specified analysis of the EMPACT‐MI trial
Source: Eur J Heart Fail. 2024 Dec 26;27(3):577–88. doi: 10.1002/ejhf.3548 (PMC11955319; doi:10.1002/ejhf.3548)

Outcome	*n*/*N*	Events/ 100 PY	Absolute risk difference	HR or RR (95% CI)	*p*-value
**Primary endpoint and components**					
**Primary composite outcome of time to first HHF or all-cause mortality**					
Normoglycaemia	30/552	4	Ref.	Ref.	
Pre-diabetes	39/447	6	4.67	1.52 (0.94–2.46)	0.0879
T2DM	130/1085	9	7.01	1.78 (1.19–2.68)	0.0051
Unknown	99/1178	6	2.90	1.34 (0.89–2.03)	0.1660
**Time to first HHF**					
Normoglycaemia	16/552	2	Ref.	Ref.	
Pre-diabetes	26/447	4	6.77	2.01 (1.07–3.76)	0.0301
T2DM	65/1085	4	5.01	1.77 (1.01–3.11)	0.0449
Unknown	46/1178	3	1.68	1.27 (0.71–2.27)	0.4124
**All-cause mortality**					
Normoglycaemia	17/552	2	Ref.	Ref.	
Pre-diabetes	18/447	3	0.64	1.19 (0.61–2.32)	0.6144
T2DM	80/1085	5	3.05	1.85 (1.08–3.15)	0.0246
Unknown	63/1178	4	1.37	1.40 (0.81–2.40)	0.2286
**Key secondary endpoints**					
**Total number of HHF or all-cause mortality**					
Normoglycaemia	39/552	5	Ref.	Ref.	
Pre-diabetes	55/447	8	3.75	1.79 (0.96–3.34)	0.0660
T2DM	170/1085	12	6.95	2.47 (1.46–4.18)	0.0008
Unknown	121/1178	7	2.14	1.45 (0.85–2.48)	0.1718
**Total number of non-elective CV hospitalization or all-cause mortality**					
Normoglycaemia	98/552	14	Ref.	Ref.	
Pre-diabetes	104/447	17	2.92	1.20 (0.80–1.81)	0.3774
T2DM	300/1085	22	7.39	1.51 (1.08–2.12)	0.0170
Unknown	228/1178	14	–0.10	0.99 (0.70–1.40)	0.9682
**Total number of non-elective all-cause hospitalization or all-cause mortality**					
Normoglycaemia	172/552	25	Ref.	Ref.	
Pre-diabetes	173/447	29	3.22	1.13 (0.81–1.57)	0.4842
T2DM	458/1085	33	7.10	1.28 (0.97–1.69)	0.0830
Unknown	335/1178	21	–4.31	0.83 (0.63–1.10)	0.1981
**Total number of hospitalization for MI or all-cause mortality**					
Normoglycaemia	31/552	5	Ref.	Ref.	
Pre-diabetes	30/447	4	–0.22	0.95 (0.49–1.85)	0.8856
T2DM	125/1085	9	4.10	1.89 (1.12–3.20)	0.0180
Unknown	88/1178	5	0.85	1.18 (0.69–2.03)	0.5375
**Other secondary endpoints**					
**Time to CV mortality**					
Normoglycaemia	11/552	1	Ref.	Ref.	
Pre-diabetes	12/447	2	0.64	1.29 (0.57–2.94)	0.5448
T2DM	68/1085	4	3.70	2.63 (1.37–5.03)	0.0035
Unknown	40/1178	2	1.04	1.48 (0.75–2.90)	0.2572
**Exploratory endpoints**					
**Total HHF**					
Normoglycaemia	22/552	2	Ref.	Ref.	
Pre-diabetes	37/447	5	3.00	2.47 (1.19–5.13)	0.0150
T2DM	90/1085	5	2.95	2.45 (1.29–4.64)	0.0061
Unknown	58/1178	3	0.57	1.28 (0.66–2.46)	0.4636

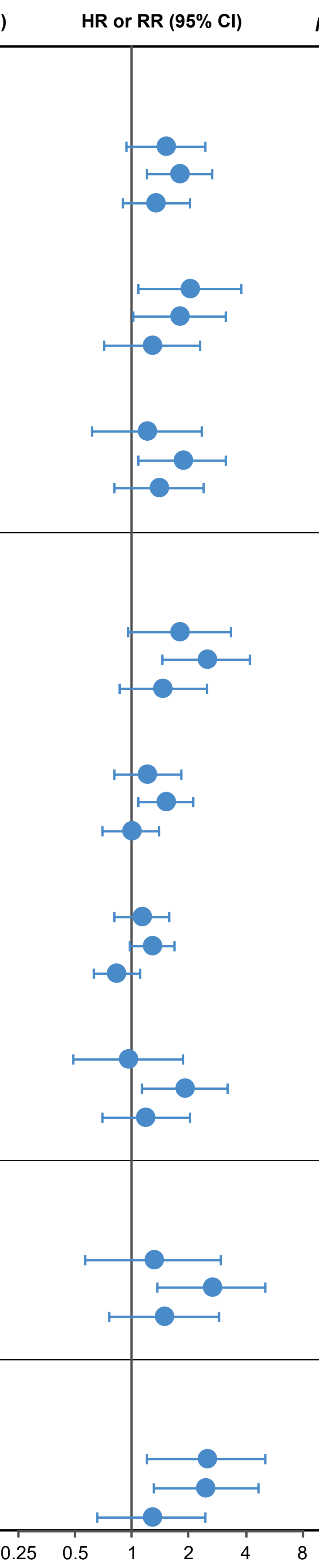

Supplement: Supplementary file 4 — Supplementary Figure S4. Effect of empagliflozin according to baseline glycaemic status (including prediabetes). Hazard ratios and Rate Ratios based on Cox regression or Negative binomial regression models adjusted for age, sex, estimated glomerular filtration rate (assessed categorically using the CKD‐EPI formula <45 vs 45–<60 vs 60–<90 vs ≥90 mL/min/1.73 m2), geographical region, type 2 diabetes, persistent/permanent atrial fibrillation, prior MI, peripheral artery disease, smoking status and LVEF (categorical or continuous). T2DM is defined as diagnosed T2DM (investigator‐reported) and undiagnosed T2D (i.e. baseline HbA1c > =6.5%). n number of patients with event (for time to first event endpoints) or number of events (for total number of events endpoint) based on N number of patients at risk. Pre‐diabetes is defined as HbA1c ≥5.7% (≥39 mmol/mL) and <6.5% (<48 mmol/mol) measured during the index presentation excluding those with baseline diagnosed T2DM. Unknown refers to no T2DM without HbA1c measured. [file EJHF-27-577-s001.pdf]
