# Supplementary material for: Empagliflozin in acute myocardial infarction in patients with and without type 2 diabetes: A pre‐specified analysis of the EMPACT‐MI trial
Source: Eur J Heart Fail. 2024 Dec 26;27(3):577–88. doi: 10.1002/ejhf.3548 (PMC11955319; doi:10.1002/ejhf.3548)
Supplement: Supplementary file 5 — Supplementary Table S1. Baseline characteristics according T2DM status, and treatment assignment to empagliflozin or placebo. Supplementary Table S2. Diabetes therapies at baseline and at discharge according to T2DM status. [file EJHF-27-577-s002.docx]

**Supplementary table 1 –** **Baseline characteristics according T2DM status, and treatment assignment to empagliflozin or placebo**

|  |  | | | | | |
| --- | --- | --- | --- | --- | --- | --- |
|  | T2DM | | No T2DM | | No T2DM and unknown HbA1c | |
|  | Empagliflozin (n=1080) | Placebo (n=1085) | Empagliflozin (n=912) | Placebo (n=999) | Empagliflozin  (n=1268) | Placebo (n=1178) |
| Characteristic* |  |  |  |  |  |  |
| Age (years) | 64 (10) | 64 (10) | 63 (11) | 64 (11) | 63 (11) | 64 (11) |
| Sex (%) |  | | | | | |
| Male | 70 | 71 | 78 | 77 | 77 | 77 |
| Female | 30 | 29 | 22 | 23 | 23 | 23 |
| Type of MI (%) |  |  |  |  |  |  |
| STEMI | 68 | 69 | 79 | 79 | 78 | 74 |
| NSTEMI | 32 | 31 | 21 | 21 | 22 | 26 |
| Race (%) |  | | | | | |
| White | 83 | 81 | 78 | 79 | 89 | 90 |
| Black or African American | 1 | 2 | 1 | 1 | 2 | 1 |
| Asian | 15 | 16 | 16 | 15 | 9 | 8 |
| Other | 0.1 | 0.4 | 0.7 | 0.2 | 0.2 | 0.1 |
| Medical history (%) |  | | | | | |
| Hypertension | 80 | 80 | 63 | 63 | 65 | 66 |
| COPD | 6 | 5 | 5 | 5 | 5 | 6 |
| AF | 10 | 11 | 11 | 11 | 11 | 11 |
| Previous stroke or TIA | 5 | 7 | 3 | 5 | 4 | 4 |
| Previous MI | 15 | 16 | 9 | 11 | 11 | 14 |
| Previous PCI | 16 | 15 | 11 | 13 | 11 | 13 |
| Previous CABG | 3 | 3 | 1 | 2 | 0.6 | 2 |
| Smoking status (%) |  | | | | | |
| Never | 27 | 26 | 25 | 29 | 26 | 27 |
| Current | 30 | 29 | 39 | 37 | 35 | 35 |
| Former | 43 | 44 | 36 | 35 | 39 | 39 |
| NT-proBNP highest (median (IQR), pg/mL)* | 1657  (580, 3097) | 1859 (728, 3962) | 1893  (759, 3576) | 1726  (717, 3663) | 1844  (925, 3756) | 2024 (717, 3620) |
| eGFR (mL/min/1.73 m^2^) | 75 (21) | 74 (22) | 78 (18) | 78 (18) | 76 (20) | 76 (20) |
| eGFR <60 mL/min/1.73 m^2^ (%) | 26 | 27 | 19 | 18 | 22 | 23 |
| Creatinine, mg/dL | 1.0 (0.3) | 1.0 (0.4) | 1.0 (0.2) | 1.0 (0.3) | 1.0 (0.3) | 1.0 (0.3) |
| Haemoglobin, g/dL | 13 (2) | 13 (2) | 14 (2) | 14 (2) | 14 (2) | 14 (2) |
| BMI, kg/m^2^ | 29 (5) | 29 (5) | 27 (5) | 27 (5) | 27 (5) | 27 (5) |
| Systolic BP, mmHg | 123 (15) | 123 (16) | 117 (14) | 118 (15) | 121 (14) | 121 (15) |
| Diastolic BP, mmHg | 74 (10) | 74 (10) | 72 (10) | 73 (10) | 74 (10) | 74 (10) |
| Medical therapy  at Baseline (%) |  | | | | | |
| Beta blockers | 78 | 78 | 79 | 82 | 75 | 73 |
| ACEi or ARB | 71 | 66 | 70 | 71 | 67 | 65 |
| ACEi/ARB/ARNI | 76 | 71 | 75 | 77 | 70 | 67 |
| MRA | 37 | 41 | 42 | 43 | 38 | 37 |
| Loop or high-ceiling diuretic | 41 | 39 | 29 | 29 | 34 | 31 |
| Medical therapy  at Discharge (%) |  | | | | | |
| Beta blockers | 88 | 88 | 86 | 89 | 86 | 85 |
| ACEi or ARB | 79 | 75 | 74 | 78 | 77 | 76 |
| ACEi/ARB/ARNI | 84 | 83 | 82 | 85 | 81 | 81 |
| MRA | 45 | 48 | 49 | 51 | 48 | 47 |
| Loop diuretic | 46 | 43 | 32 | 31 | 41 | 36 |

Where not stated, data present mean (standard deviation).
*Based on 422 patients in empagliflozin and 381 patients in placebo groups with T2DM, 458 patients in empagliflozin and 540 patients in placebo groups with no T2DM, 377 patients in empagliflozin and 370 patients in placebo groups with unknown HbA1c.

**Supplementary Table 2 – Diabetes therapies at baseline and at discharge according to T2DM status**

|  | T2DM (n=2165) |
| --- | --- |
| **Baseline** |  |
| Metformin | 47% |
| Sulfonylureas | 14% |
| DPP-4 inhibitor | 8% |
| GLP1 receptor agonist | 3% |
| Insulin | 23% |
| **Discharge** |  |
| Metformin | 53% |
| Sulfonylureas | 15% |
| DPP-4 inhibitor | 9% |
| GLP1 receptor agonist | 4% |
| Insulin | 24% |
